# Review of the 2020 ESC Guidelines for the Diagnosis and Management of Atrial Fibrillation—What Has Changed and How Does This Affect Daily Practice

**DOI:** 10.3390/jcm10173922

**Published:** 2021-08-30

**Authors:** Johanna B. Tonko, Matthew J. Wright

**Affiliations:** 1 Department of Cardiology, Guy’s and St. Thomas’ NHS Foundation Trust, London SE1 7EH, UK; matthew.wright@kcl.ac.uk; 2 Faculty of Life Sciences and Medicine, King’s College London, London SE1 1UL, UK

**Keywords:** atrial fibrillation, atrial cardiomyopathy, rate control, rhythm control, catheter ablation, anti-arrhythmic drugs, stroke, heart failure

## Abstract

The high prevalence of atrial fibrillation (AF) in the overall population and its association with substantial morbidity, increased mortality and health care cost has instigated significant basic and clinical research efforts over recent years. The publication of multiple new high-quality randomized multi-center trials in the area of AF management and the rapidly evolving technological progress in terms of diagnostic possibilities and catheter ablation in recent years demanded a revision of the previous ESC AF Guidelines from 2016. The 2020 guidelines provide up-to-date, evidence-based guidance for the management of AF. One of the most important innovations is the presentation of a new concept for structural characterization of AF (the “4S AF scheme”) replacing the traditional classification based on its temporal pattern alone (paroxysmal-persistent-permanent). The 4S-AF-scheme highlights the importance of systematic assessment of stroke risk, severity of symptoms, total AF burden and underlying substrate as the foundation for effective and individualized AF treatment for each and every patient. Further novelties relate to the presentation of an easy and intuitive management pathway (“ABC pathway”) and strengthening the recommendations for early rhythm control, in particular the role of first line catheter ablation in heart failure. Another core component of the guidelines is the focus on patient involvement to achieve optimal outcomes. Patient education, shared decision making and incorporation of patient values and patient reported outcome of treatment interventions as well as integrated care by a multidisciplinary team all have a central role in the proposed management pathway for AF.

## 1. Introduction

Globally atrial fibrillation (AF) is the most common cardiac arrhythmia in adults and the number of patients affected continues to rise with a currently estimated life time risk of 1 in 3 [1]. This is partly secondary to overall ageing of the population and survival of patients with other arrhythmogenic cardiac disease but also due to the improved diagnostic possibilities, including widely available screening via smartwatches, smartphones and continuous monitoring methods, such as implantable loop recorders. The detrimental effect of AF on morbidity (including stroke, heart failure, cognitive decline and depression) and mortality has been described. The combination with its high prevalence has let to intense efforts to improve its management and thereby outcome for the individual patient and to reduce strain on the healthcare system.

The last decade has seen significant advances in the understanding of the mechanism of AF and its treatment. The ESC Guidelines 2020 [2]. provide a summary of the rapidly growing evidence in regards to AF and offer a compact up-to-date evidence-based guidance for screening, work-up and management of this complex arrhythmic disorder with nearly 1500 references. In addition to the general recommendations, the guidelines also dedicate a chapter to AF in special patient groups, including pregnancy, congenital heart disease or inherited cardiac conditions or in the setting of stroke, active bleeding and the postoperative period.

## 2. Screening and Diagnosis

### 2.1. Novel Screening Tools and Their Integration in Clinical Practice

Early identification and commencement of appropriate treatment for AF may prevent potentially deleterious consequences such as stroke and heart failure as well as progression of the underlying substrate. Despite the intuitive usefulness of AF screening hard evidence from randomized studies supporting a prognostic benefit and cost-effectiveness of AF screening has long been lacking. The 2020 guidelines recommend systematic screening for patients aged >75 years or those at high risk of stroke (IIaB) [3] and opportunistic screening in patients >65 years (IB) [4,5]. A new recommendation in 2020 represents the opportunistic screening of patients with hypertension or obstructive sleep apnea. New results of the 5-year follow up of the STROKESTOP study [6] presented at the European Heart Rhythm Association (EHRA) 2021 congress [7] strengthen the argument for systematic screening in the elderly of 75 years demonstrating a net clinical benefit. Further large-scale AF screening studies investigating hard endpoints including stroke reduction and bleeding are expected in the upcoming years including the GUARD AF (recruiting) [8], SAFER study [9] and the LOOP Study (recruitment completed) [10].

Mobile health technologies have revolutionized AF screening with a multitude of commercially and medically available wearable monitors [11] in addition to the rising number of patients with implantable loop recorders and cardiac implantable electronic devices (CIED). Particular ECG monitoring via smartwatches and phones has the potential for large-scale population-based screening due to their widespread use (exemplified by the Apple Heart Study recruiting over 400,000 patients in only 8 months [12]). Only few of commercially available wearable monitors are investigated for medical use and signal quality and automatic algorithms vary. However, overall diagnostic accuracy and performance of automated algorithms appear to be acceptable [13]. The 2020 ESC Guidelines encourage the use of new wearable, commercially available single or multiple lead ECG monitors (including photo-plethysmographs on smartphones and smart watches with or without dedicated connectable devices) as AF screening tools as long as the significance and the treatment implications in case of detecting AF have been discussed with the patient. Yet, there is no recommendation on the appropriate interval and duration of AF screening with these devices. Additionally, for the definite diagnosis of AF the verification of the ECG tracing recording over at least 30 s by a physician with expertise in ECG is still required. The latter remains essential to avoid misinterpretation, leading to overdiagnosis, exposure to unnecessary further tests and overtreatment. 

A future development in AF screening to look out for will be the application of machine learning and artificial intelligence to sinus rhythm ECGs to identify patients with paroxysmal AF [14]. 

### 2.2. Management of Atrial High Rate Episodes (AHRE) and Subclinical Atrial Fibrillation in CIED

For patients with cardiac devices with automated continued monitoring and tracing storage, regular device interrogation to screen for atrial arrhythmias is recommended. 

Atrial high-rate episodes (AHRE) are most commonly defined as paroxysmal episodes of atrial rates >175–180 bpm lasting >5–6 min detected by CIEDs. If visual inspection of the intracardiac electrogram confirms the presence of AF, it may be referred to as “subclinical atrial fibrillation”. It is recommended that these patients undergo screening for risk factors and co-morbidities associated with AF, but it remains controversial which exact burden of AHRE and subclinical AF mandates medical treatment. It is unknown whether any rate or rhythm control intervention at this stage may prevent progression to clinical AF and no recommendation in regards to their initiation are given. 

The need and benefit of anticoagulation in AHRE is currently investigated by two ongoing large scale randomized trials (ARTESIA trial, identifier NCT01938248 expected study completion date December 2022 [15], and NOAH trial, identifier NCT02618577, expected study completion data March 2022 [16]). Pending the results of these randomized trials, the ESC guidelines 2020 remain in line with the EHRA consensus document “Management of Device detected Subclinical Atrial Arrhythmias”, published in 2017 [17], stating that anticoagulation may be considered in patients with higher risk of stroke (CHA_2_DS_2_-VASc ≥ 2 in men and ≥3 in women) and AHRE durations >24 h. The cut off of >24 h is supported by large-scale trials, such as the ASSERT trial [18], demonstrating an increased risk of ischemic stroke or systemic embolism with AHRE episodes >24 h. For rare, asymptomatic, short-lived episodes, a watch-and-wait strategy with regular reassessment for an increase in the AHRE burden and risk of stroke is proposed.

## 3. Structured Characterization of Atrial Fibrillation

**Figure 1:**
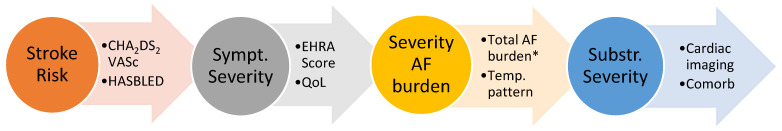
Structured characterization of AF: the newly introduced “4S-AF scheme” of the ESC Guidelines 2020 incorporates four domains to assess the individual disease state of the patient and highlights areas with essential therapeutic and prognostic implications. For a detailed explanation, see text. *Total AF burden = total time in AF per monitoring period or the longest episode or number of episodes.

One of the major changes of the new 2020 Guidelines is the paradigm shift away from a simple AF *classification* towards a *structured characterization* to address specific domains with treatment and prognostic implications in order to understand the individual disease state of the patient (summarized in Figure 1). It also allows to streamline the assessment of AF patients in view of the growing complexity of the management and acknowledges the association of atrial fibrillation with multiple other cardiovascular and extracardiac diseases influencing its progression and prognosis. 

**Figure 2:**
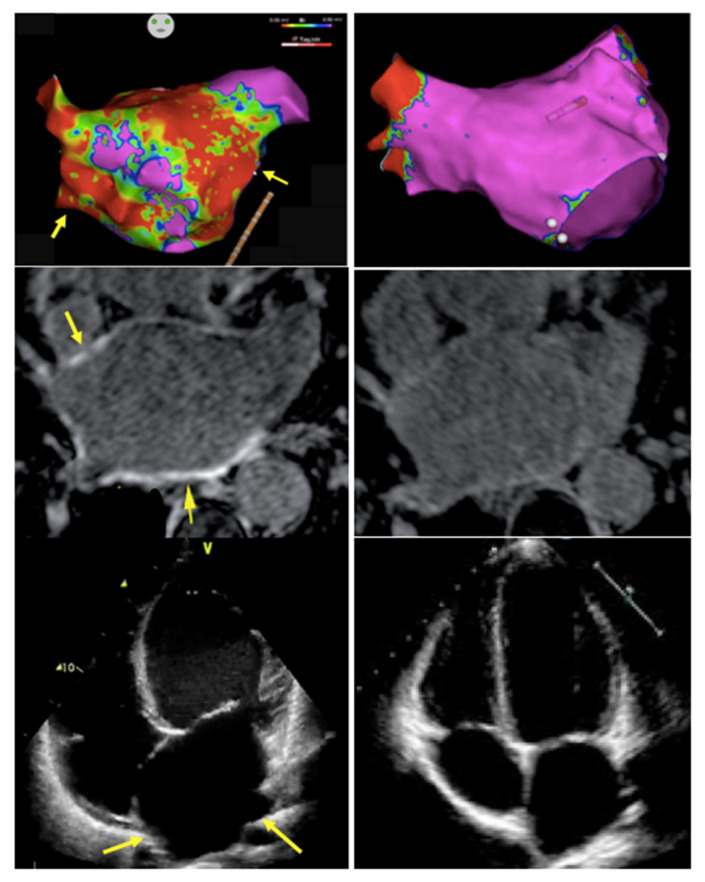
Substrate Severity: Multimodality assessment of the underlying atrial substrate to guide individualized treatment strategies. Figure 2 demonstrates three examples of severely diseased left atria compared to structurally normal ones. (**Top row**): Electro-anatomical voltage map of the left atrium: (**Left**) Left atrium with extensive scarring (red areas corresponding to bipolar voltage <0.05 mV, indicating scarring); (**Right**) Left atrium with normal “healthy” bipolar Voltage >0.5 mV (pink areas). (**Middle row**) Atrial CMR images: (**Left**) late gadolinium enhancement (yellow arrows) within the anterior and posterior wall of the left atrium indicating fibrosis; (**Right**) no detectable LGE. (**Bottom row***)* TTE Images Apical 4-chamber view: (**Left**) significantly enlarged left atrium; (**Right**) normal-sized left atrium.

The 4S AF scheme includes a comprehensive cardiovascular assessment including the following domains:

- Stroke Risk: As in previous guidelines, the recommended assessment tool for stroke risk estimation remains the well-known CHA_2_DS_2_-VASc Score. The ESC taskforce has refined some of the risk factors by adding precise blood pressure and blood glucose cut-offs as well as including hypertrophic cardiomyopathy, heart failure with preserved ejection fraction and asymptomatic moderate to severe LV dysfunction into the score. Opposed to the original score, angiographically documented significant coronary artery disease (regardless of symptom status) is now included as well. The temporal pattern and total burden of AF are not part of the stroke risk assessment in the current guidelines.
- Symptom Severity: The severity of symptoms should be evaluated in a standardized manner with the EHRA symptom score ranging from 1 to 4 or via quality-of-life questionnaires. Importantly, a symptom-rhythm correlation should be established to differentiate from symptoms due to underlying co-morbidities.
- Severity of AF burden: Assessing the AF burden includes not only the traditionally used classification of the temporal pattern into paroxysmal, persistent and permanent AF but also the total AF burden defined as the percentage of time in AF for a defined time frame. Higher AF burden have been associated with higher stroke risk [19] and mortality rates (if >6–24 h of AF per week) [20], poorer response to rhythm control therapy [21] and may represent progression of advanced atrial remodeling [22]. However, it remains unclear whether progressive AF burden is primarily a marker or a driver or both of progression of the underlying disease and adverse prognosis.
- Substrate Severity: A growing body of evidence showed that the severity and extent of left atrial structural and electrical remodeling has prognostic value for patients with AF. Technological improvements in non-invasive imaging modalities (echocardiography with TDI and strain, cardiac MRI with Late-Gadolinium Enhancement (LGE), cardiac CT) as well as invasive high density electro-anatomical contact mapping has allowed for more detailed assessment of the underlying substrate of AF. Nowadays, it is commonly acknowledged that left atrial size alone is not able to accurately define the disease state. Atrial wall fibrosis [23] and wall thickness [24], epicardial fat infiltration [25], atrial conduction velocities [26] or geometrical assessments such as sphericity [27] are a number of further parameters that have shown prognostic value and may guide treatment decisions, though most are not yet routinely assessed in daily practice. The guidelines suggest the assessment of atrial electrical and mechanical dysfunction and thrombotic risk by means of multimodality imaging and biomarkers as well as comprehensive review of cardiovascular risk factors and comorbidities affecting the atrial substrate. Figure 2 illustrates examples of normal compared to diseased left atrial substrate assessed by electro-anatomical mapping, MRI and TTE.

## 4. Treatment: The ABC Pathway

**Figure 3:**
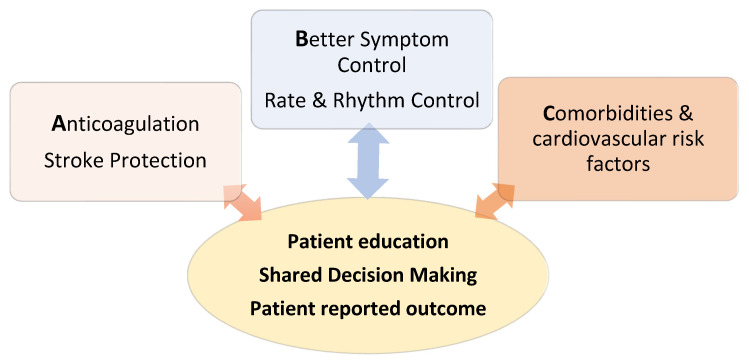
Illustration of the components of the ABC of AF Management in 2020 to ensure effective AF management for each patient. The three treatment pillars should be incorporated in an integrated care concept, which has patient education, shared decision making and patient reported outcome at its core.

To counterbalance the growing complexity of AF management due to a multiplicity of available treatment options, the 2020 guidelines have introduced an easy intuitive treatment algorithm to ensure effective and appropriate AF management for all patients: the ABC pathway (summarized in Figure 3). This pathway is incorporated into the overlying concept of “integrated care” delivered by an interdisciplinary team. The core of this approach is patient education, shared decision making, consideration of patient values when discussing treatment options and, if implemented, an assessment of “patient-reported outcome” measures. Every domain of the mosaic that constitutes a contemporary structured AF management should be addressed to achieve the best clinical result for the individual patient.

### 4.1. Anticoagulation

Optimal stroke protection remains at the heart of AF management. Oral anticoagulation can reduce risk of ischemic stroke by 65% at the cost of an increase of hemorrhagic stroke of 0.3%/year [28]. Consistent with the 2016 guidelines every patient with at least a CHA_2_DS_2_-VASc score of 1 in men or 2 in women should be considered for anticoagulation (IIa B) and men with a score of ≥2 or women ≥3 have a clear I A recommendation. The guidelines emphasis the dynamic nature of many risk factors, and hence, all patients, particularly those with initially low scores, should be periodically re-evaluated. Despite the development of a new risk score, including several other clinical risk factors, imaging and biomarkers not included in the CHA_2_DS_2_-VASc score, the latter continues to have the best evidence for predicting thromboembolic risk [29]. Initiation and continuation of anticoagulation should be routinely accompanied by a bleeding risk assessment (HAS-BLED) and, if present, treatment of modifiable bleeding risk factors. Importantly, a high bleeding risk score per se should not lead to withholding anticoagulation.

A clear preference is given to new oral anticoagulants (Rivaroxaban, Edoxaban, Apixaban, Dabigatran) over Vitamin K antagonists given the compelling evidence for better safety and efficacy with overall statistically significant mortality reduction compared to Warfarin [30]. Exempt from this recommendation are patients with mechanical heart valves or moderate–severe mitral stenosis where NOACs remain contraindicated. 

In case of absolute contraindications to anticoagulation, a left atrial appendage occlusion may be considered, but the class of recommendation remains IIbB as in 2016. The concept of LAA occlusion is limited by acknowledging that AF acts also as a risk marker of stroke risk and mechanical LAA occlusion may, therefore, only be a partial substitute for anticoagulation. Incomplete occlusion may even increase the risk for thrombus formation. Additionally, current commercially available percutaneous devices (WATCHMAN and AMULET) require a period of dual antiplatelet therapy with Aspirin and Clopidogrel for 1–6 months to avoid device related thrombus during re-endothelialization followed by long-term aspirin monotherapy. At the time of the guideline publication, available randomized outcome data for percutaneous LAA occlusion were limited to the Watchman device, but further studies are currently in progress. The randomized multi-center open-label COMPARE LAAO trial comparing the Watchman and other LAA occlusion devices to usual care of antiplatelet therapy or nothing started recruiting in January 2021 [31]. In 2021, new evidence (LAAOS III trial) has been published regarding the benefit of surgical LAA occlusion in addition to ongoing anticoagulation. The trial demonstrated significantly reduced rates of ischemic stroke with surgical LAA occlusion at time of cardiac surgery for other reasons in patients with AF [32].

A special challenge represents anticoagulation in the context of acute (ACS) or chronic coronary syndrome (CCS) requiring percutaneous coronary intervention. In general, the recommendations for duration of triple therapy have been shortened compared to previous guidelines. For uncomplicated PCIs, early cessation (≤1 week) of aspirin and continuation of dual therapy with oral anticoagulation and a P2Y12 inhibitor (preferably Clopidogrel) for 6 to 12 months (ACS) [33,34,35,36] or 3 to 6 months (CCS) [37,38,39] are recommended. In patients at high ischemic and low bleeding risk, longer duration of dual therapy can be considered. Equally extended triple therapy with Aspirin, Clopidogrel and an oral anticoagulation for longer than 1 week after an ACS should be considered when risk of stent thrombosis outweighs the bleeding risk, with the total duration ≤1 month. 

### 4.2. Better Symptom Control

The second pillar of the ABC pathway refers to symptom control by means of rate or rhythm management with medication, cardioversion or invasive therapies. Elimination or amelioration of symptoms has always been one of the major driving forces for therapy. However, the positive effect of restoring and maintaining sinus rhythm goes beyond symptom control and has been shown to also increase exercise capacity [40] and quality of life [41], improve ventricular ejection fraction [42] as well as reduce left atrial size [43], atrial arrhythmia burden and cardiac hospitalizations [44] and most recently also mortality [45]. With rate control alone, atrial substrate may advance over time, despite adequate ventricular rate to the point where successful rhythm control might no longer be feasible.

Table 1 gives an overview of the ESC 2020 recommendations for medical rate and rhythm control options.

#### 4.2.1. Rate Control

In terms of rate control, no significant changes have been implemented compared to the 2016 Guidelines. It remains part of the general background therapy for all AF patients, particularly for those with failed or contraindicated rhythm control attempts. The optimal target heart rate remains unclear, but generally, an initial lenient heart rate control of <110 bpm is accepted, with a more stringent control recommended in case of persistent symptoms or interference with cardiac resynchronization therapy.

Rate control can be achieved by means of a mono- or combination therapy of betablocker and/or non-dihydropyridine calcium channel blocker and/or digoxin. Previous findings from observational studies of excess mortality of digoxin use in atrial fibrillation patients [46,47] were attributed to a selection and prescription bias [48]. A pace-and-ablate strategy with AV node ablation should be considered in medically uncontrolled heart rates (IIaB). Amiodarone as rate control may only be considered as a third line agent if other options failed or are unavailable.

It should be noted that drugs traditionally used for rate control did not show prognostic benefit for heart failure patients with reduced ejection fraction. A meta-analysis in 2014 found that betablocker did not provide prognostic benefit in the presence of AF [49] and no new high quality randomized data has been published since. Non-dihydropyridine calcium channel blockers are generally contraindicated in LVEF <40%. The impact of cardiac glycosides in HFrEF is currently being revisited in the ongoing DIGIT HF trial [50] in a contemporary patient cohort with sinus rhythm or atrial fibrillation. 

#### 4.2.2. Rhythm Control

Despite optimal management for AF, including anticoagulation and optimal rate control, patients still suffer from stroke, coronary syndromes, heart failure and cardiovascular death at a rate of approximately 5% per year [51,52]. Data from large registries suggested additional benefits of rhythm control (e.g., reduced rate of death, stroke, myocardial infarction and HF hospitalization [53] as well as dementia [54,55]). However, given the observational nature of the registry data, questions remained whether sinus rhythm may be just a marker of a healthier heart and, hence, a better outcome or whether maintenance was adding to this improved outcome. Only recently randomized controlled studies for heart failure [55] and non-heart failure patients [56,57] were able to show a net prognostic benefit for rhythm control independent of symptom status. 

The 2020 Guidelines were published prior to the seminal EAST AFNET 4 study [58] and base the indication for rhythm control (in none heart failure patients) primarily on the symptom status of the patient. For various reasons this merits further discussion.

##### New Evidence for the Prognostic Benefit of Rhythm Control

The assumption that rate and rhythm control were equal in terms of prognosis was based on multiple large randomized studies from the early 2000s comparing medical rhythm vs rate control in non-heart failure patients [56] and heart failure patients [57]. In part, this has been attributed to the practice of stopping anticoagulation after successful rhythm control in these trials, the per se lower success rates of antiarrhythmic drugs compared to catheter ablation, as well as their pro-arrhythmogenicity and side effects, particularly if used in combination, all of which might have offset the benefits or rhythm control for AF. An on-treatment analysis of the AFFIRM study, one of the landmark trials investigating medical rate versus rhythm control, subsequently showed that the two predictors of reduced mortality were the maintenance of sinus rhythm and the use of warfarin [58].

Two large prospective multi-center randomized trials have dominated the debate of rhythm control in AF in the last years: the CABANA trial [59,60] from 2019 (catheter ablation versus medical treatment in paroxysmal and persistent AF, including longstanding) and EAST AFNET 4 study [61,62,63] in 2021 (rate versus early rhythm control by means of AAD or catheter ablation for recently diagnosed AF <1 year). The CABANA trial failed to demonstrate prognostic benefit in the intention-to-treat analysis. However, crossover rate to the catheter ablation group was 27% and the treatment-received analysis showed significantly improved outcome with invasive rhythm control. The EAST AFNET 4 study reached a positive primary endpoint, confirming the clinical benefit of early rhythm control with a significant reduction of the composite endpoint of cardiovascular death, stroke, HF hospitalization and ACS including for patients above 75 years of age. Of note, 30% of the patients were asymptomatic at baseline and symptom control was equally good in the rate and rhythm control group. Importantly, both trials revealed an excellent safety profile of rhythm control for antiarrhythmic drugs as well as catheter ablation. 

##### Optimal Timing of Rhythm Control

The importance of timing of rhythm control initiation in reference to the time of diagnosis is still under debate, and it should likely not be the only decisive factor. Complete characterization of the AF disease state of the individual patient is the cornerstone for decision making regarding rhythm control. However, evidence is building up to support early rhythm control approaches. The findings of EAST AFNET4 and other recently published randomized trials with good results obtained for catheter ablation as first line treatment in paroxysmal AF [60,61,62] as opposed to the neutral endpoint in the CABANA trial, which included longstanding persistent AF patients, strengthen the argument for early initiation in the disease process. A meta-analysis from 2020 showed an unsurprisingly improved likelihood of procedural success for AF with shorter duration (<1 year) between first diagnosis and ablation [63], whereas ablation in patients with longstanding persistent AF over 2 years yield lower success rates [64]. This approach is also pathophysiologically supported. High burden of atrial fibrillation is associated with progressive left atrial structural remodeling (dilatation, wall thickening and fibrosis), whereas AF ablation may result in reverse remodeling. This should be taken into account for timing ablative interventions [65,66,67,68].

##### Choice of Rhythm Control Modalities

Various options to achieve rhythm control are available and can be used complementarily. The pros and cons of the modalities need to be discussed with the patient to allow for an informed decision based on their preference as well as on the clinical profile. 

Cardioversion by means of synchronized electrical shocks remains the first-line treatment for hemodynamically unstable patients, whereas hemodynamically stable patients may be treated with electrical or pharmacological (Flecainide, Propafenone, Vernakalant, Amiodarone or Ibutilide) cardioversion. A new recommendation in the 2020 guidelines involves a “wait-and-watch” delayed-cardioversion strategy for recent onset symptomatic atrial fibrillation. This strategy has been shown to be non-inferior to early cardioversion in achieving a return to sinus rhythm, with a spontaneous conversion rate of 69% within 48 h [69,70]. 

For long-term pharmacological rhythm control, no new antiarrhythmic drugs have been introduced and the options in 2020 remain limited to class I (Flecainide, Propafenone) or III (Amiodarone, Dronedarone, Sotalol) drugs. Yet, better understanding of their proarrhythmogenic potential in certain patient groups (structural heart disease, conduction system disease), avoidance of combination of antiarrhythmic drugs and implementation of appropriate monitoring of proarrhythmic factors has improved the safety profile of AADs in daily practice, as demonstrated in large scale trials [71,72]. The 2020 Guidelines highlight the extracardiac toxicity of Amiodarone which should be reserved as second- or third-line treatment.

Catheter ablation has become a well-established, safe and superior alternative to antiarrhythmic drugs for restoration and maintenance of sinus rhythm and symptom control with multiple randomized trials in paroxysmal AF [67,68,69,70] as well as persistent AF [71], supporting this approach. A novelty in the 2020 guidelines is the incorporation of risk factors for AF recurrence (including hypertension, obesity, metabolic syndrome and sleep apnea) into the decision making for catheter ablation. However, as mentioned above for non-heart failure patients, symptom status remained the decisive factor for referral for catheter ablation in the guidelines. In light of the EAST-AFNET4 trial demonstrating benefits beyond symptom improvement and the generally improved safety profile and shortened procedure times of AF catheter ablations, this will require further discussion and catheter ablation is likely to take a more prominent role in the future. 

In drug-refractory AF and failed percutaneous ablation, hybrid approaches combining endo- and minimal invasive epicardial ablation have become an increasingly employed option and should be considered (IIaB). In 2020, the CONVERGE trial demonstrated successful rhythm control of longstanding persistent AF with maintenance of sinus rhythm in 67% at 1 year by means of combined epi- and endocardial ablation [72].

##### Focus on AF and Heart Failure

Catheter ablation was demonstrated not only to be feasible and safe in heart failure [73,74] but has also been associated with positive outcomes for symptoms, reduction in left atrial size [75] and improvement in LVEF [76], peak VO2 [77] and BNP [78] compared to rate control or medical rhythm control [79]. In 2019, the CASTLE AF study [80] and the heart failure subgroup analysis of the CABANA Study [81] did provide evidence of significant all-cause death and heart failure hospitalization reduction. On the other hand, based on the findings of the negative AMICA trial [75] (persistent AF with LVEF <35%), it has been suggested that the benefit of AF ablation may be affected by the extent of HF at baseline with limited benefit for patients with very advanced heart failure. The impact of pre-existing structural heart disease, including scarring and fibrosis, on procedural efficacy and prognostic benefit of AF ablation in heart failure patients will be further investigated in the upcoming CAMERA-MRI II trial [76].

Rhythm control benefit in the population of HFpEF patients is less well investigated. The above-mentioned subgroup analysis of the CABANA Study included 79% of HFpEF patients and, together with observational data [77,78], suggests that rhythm control is associated with a lower risk of all-cause mortality in HFpEF. Dedicated prospective randomized studies are needed to confirm this potential benefit.

Based on the above-mentioned trials, the 2020 ESC Guidelines recommend to consider AF catheter ablation for heart failure with a reduced ejection fraction, independent of symptom status, to improve prognosis (IIa B). If atrial fibrillation with rapid conduction is the suspected cause of heart failure (“tachy-cardiomyopathy”), the class of recommendation for catheter ablation has been upgraded to I B. The CAMERA-MRI study [79] was even able to show significant benefit of AF ablation in patients with good rate control, identifying AF as an underappreciated reversible cause of left ventricular systolic dysfunction. 

##### Defining, Measuring and Predicting Rhythm Control Success

Traditionally rhythm control trials use a cut-off of >30 s of atrial arrhythmia recurrence as the definition of treatment failure. However, it has been argued that a single self-limited short-lived recurrence of atrial arrhythmia following therapy may be an overly stringent criterion for failure [82]. Assessment of total AF burden by extended or continuous heart rhythm monitoring allows for more accurate appraisal of the effect of treatment and has been suggested as a clinically more meaningful endpoint [80]. Other more “liberal endpoints” or “patient defined outcome endpoints” have also been proposed [81]. The 2020 ESC guidelines already strongly recommend to assess patient-reported outcome after treatment interventions. 

Multiple scores have been developed for pre-procedure prediction of AF recurrence after ablation with the aim to improve patient selection and individualize treatment to reduce recurrence rate. A recent metanalysis [83] found 13 models, with no model providing consistently good discriminatory ability across the studies. Additionally, no model showed consistently better discrimination compared to others. The most commonly used model variables were left atrial parameters, type of AF and age, and to a lesser extent gender and eGFR. An open discussion about recurrences and risk of need for repeated catheter ablation should be an integral part of the shared decision making with the patient. 

## 5. Cardiovascular Risk Factors and Comorbidities

The third pillar of the ABC pathway relates to systematic assessment, identification and aggressive treatment of all cardiovascular risk factors and comorbidities associated with AF. Isolated management of specific conditions alone is often insufficient, as commonly, they are not the sole contribute to AF. 

In addition to encouraging intense lifestyle modifications to achieve normal body weight, reduce alcohol intake and increase physical activity, a particular focus is on optimal control of hypertension, sleep apnea treatment as well as addressing other well-known cardiovascular risk factors, including diabetes mellitus, hyperlipidemia and smoking. All of these have been identified to have a negative impact on the clinical course of AF and contribute to negative atrial remodeling. The guidelines reinforce the importance of patient education and involvement in order to achieve optimal outcomes.

## 6. Conclusions

The 2020 Guidelines provide a comprehensive, extensively referenced overview and guidance for the increasingly complex management of atrial fibrillation. Importantly, they emphasize the need for integrated care of AF patients and the essential role of assessment and treatment of comorbidities to achieve optimal outcome. However, the intense research in the field of AF as well as the rapid technological progress in the area of mobile health technologies and catheter ablation underline the dynamic nature of evidence-base care exemplified by the multitude of new evidence published only within the last year since the publication of the 2020 Guidelines. 

In our opinion, most importantly, the role of early rhythm control initiation in general and the superiority of catheter ablation to achieve it in particular should be highlighted to a greater extent and a more differentiated approach beyond symptom status alone should be adopted. Despite the substantial evidence in favor of catheter ablation to restore and maintain sinus rhythm as well as decreased procedural complication rates and procedure times with contemporary technologies and workflows, many referrers still reserve this valuable option as escalation treatment for patients with failed drug therapy only. Based on the available data, we emphasize the importance of early referrals to centers offering catheter ablation to evaluate patients for this still-underutilized treatment option. 

Numerous open questions in regards to AF management remain, including the optimal management of AHRE and subclinical AF as well as of very late presenting patients with significantly advanced AF. Additionally, better characterization of drivers of AF related complications (including total AF burden, structural atrial changes and comorbidities) to stratify and individualize treatment strategies (including antiarrhythmic drugs versus ablation or upfront combination, choice of energy sources of catheter ablation and ablation strategies beyond pulmonary vein isolation) will take a central role in further research efforts. 

## Figures and Tables

**Table 1:** Medical rate and rhythm control options in AF management—overview.

	Rate Control			Medical Rhythm Control ^+^
		No significant SHD	CAD, VAD, HFpEF	HFrEF
1. Line	Betablocker NDCC * (IB)	Flecainide Propafenone Dronedarone (IA)	Dronedarone Amiodarone (IA)	Amiodarone (IA)
2. Line	Digoxin (IB) Combinations (IIa)	Sotalol (IIbA)	Sotalol (IIbA)	-
3. Line	Pace&Ablate (IIaB) Amiodarone (IIbB)			

In brackets/bold letters = class of recommendation and level of evidence of ESC Guidelines 2020. * Choice of drugs based on comorbidities including HFrEF, severe COD/Asthma, pre-excited AF. ^+^ Factors favoring rhythm control: Patient’s choice, symptomatic AF, young age, first episode, tachycardia-mediated cardiomyopathy, difficult rate control, normal to moderate increased LAVI, no or few comorbidities, AF precipitated by temporary event; Abbreviations: NOAC, novel oral anticoagulants; VKA, Vitamin K antagonists; NDCC, non dihydropyridine calcium channel blocker; CAD, coronary artery disease; VHD, valvular heart disease; HFp/rEF, heart failure with preserved/reduced Ejection Fraction.

## Data Availability

No original data generated, analysed or reported.

## Abbreviations

AF	Atrial fibrillation
AHRE	Atrial High Rate Episodes
CIED	Cardiac implantable electronic devices
HFrEF	Heart failure with reduced ejection fraction
HFpEF	Heart failure with preserved ejection fraction
CAD	Coronary artery disease
VHD	Valvular heart disease
ECG	Electrocardiogram
NOAC	Novel oral anticoagulants
VKA	Vitamin K Antagonists
NDCC	Non dihydropyridine calcium channel blocker,
TTE	Transthoracic echocardiogram
LV	Left Ventricle
MRI	Magnetic Resonance Imaging
LGE	Late Gadolinium Enhancement
CTCA	Computer-tomography Coronary Angiogram
AAD	Antiarrhythmic Drugs
IST	Inappropriate Sinus Tachycardia
RFA	Radiofrequency Ablation
